# Modulation of the RNA Interference Activity Using Central Mismatched siRNAs and Acyclic Threoninol Nucleic Acids (aTNA) Units

**DOI:** 10.3390/molecules20057602

**Published:** 2015-04-24

**Authors:** Adele Alagia, Montserrat Terrazas, Ramon Eritja

**Affiliations:** 1 Institute for Advanced Chemistry of Catalonia (IQAC-CSIC), CIBER-BBN Networking Centre on Bioengineering, Biomaterials and Nanomedicine, Jordi Girona 18-26, 08034 Barcelona, Spain; E-Mails: adele.alagia@iqac.csic.es (A.A.); montserrat.terrazas@irbbarcelona.org (M.T.); 2 Institute for Research in Biomedicine (IRB Barcelona), Baldiri Reixac 10, 08028 Barcelona, Spain

**Keywords:** RNAi, siRNA, single-stranded siRNA, L-threoninol, Ago2, RISC, silencing asymmetry, wobble base pair, on-/off-target effects, central mismatched siRNA

## Abstract

The understanding of the mechanisms behind nucleotide recognition by Argonaute 2, core protein of the RNA-induced silencing complex, is a key aspect in the optimization of small interfering RNAs (siRNAs) activity. To date, great efforts have been focused on the modification of certain regions of siRNA, such as the 3'/5'-*termini* and the seed region. Only a few reports have described the roles of central positions flanking the cleavage site during the silence process. In this study, we investigate the potential correlations between the thermodynamic and silencing properties of siRNA molecules carrying, at internal positions, an acyclic L-threoninol nucleic acid (aTNA) modification. Depending on position, the silencing is weakened or impaired. Furthermore, we evaluate the contribution of mismatches facing either a natural nucleotide or an aTNA modification to the siRNA potency. The position 11 of the antisense strand is more permissive to mismatches and aTNA modification, in respect to the position 10. Additionally, comparing the ON-/OFF-target silencing of central mismatched siRNAs with 5'-terminal modified siRNA, we concluded: (i) central perturbation of duplex pairing features weights more on potency rather than silencing asymmetry; (ii) complete bias for the ON-target silencing can be achieved with single L-threoninol modification near the 5'-end of the sense strand.

## 1. Introduction

RNA interference (RNAi) is a powerful gene regulatory process that allows the inhibition of genes in a very specific way [1]. Double stranded RNAs known as small interfering RNAs (siRNAs), recognized by the RNA-Induced Silencing Complex (RISC) [2], efficiently trigger the RNAi pathway. After siRNA loading into the RISC, the sense strand is cleaved and released [3]. At this stage, the active RISC, bearing only the antisense strand, can match with the corresponding complementary messenger RNA (mRNA) sequence. The cleavage of the target mRNA mediates the inhibition of the mRNA translation into the corresponding protein.

The great therapeutic potential of blocking the expression of specific genes [4] makes the siRNA-based approach, together with other nucleic acids based technologies, such as antisense oligonucleotides, aptamers, and exon-skipping oligonucleotides, emerging drugs for the treatment of cancer diseases [5,6]. Despite the promising benefits, oligonucleotides have some disadvantages, such as poor nuclease stability, poor cellular uptake, off-target effects, and non-specific immune responses [7,8]. To overcome these important hurdles, the chemical modification strategy was widely used. For example, the introduction of sugar phosphate backbone modifications usually increases stability to nucleases [9,10,11]. In addition, some of these modifications can also affect the conformational *equilibrium* and the overall flexibility of nucleic acid duplexes [12].

**Figure 1:**
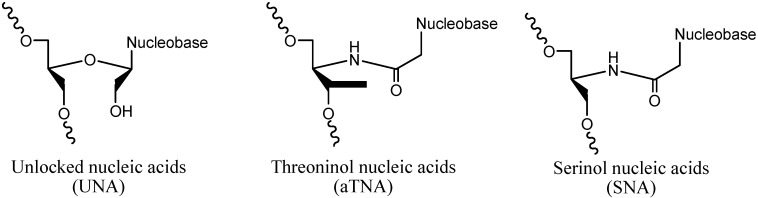
Chemical structures of flexible acyclic derivatives of nucleosides used in RNA interference experiments. Threoninol and serinol nucleic acids may be formed by D or L stereoisomers.

Hereafter, conformational restricted nucleosides, such as locked nucleic acids (LNA) [13,14], carbocyclic pseudonucleosides [15], arabino nucleic acids (ANA) [16], and 2'-F-ANA [17], imposing restriction of the conformation of the furanose system in the *North* quadrant of the pseudorotational cycle (C3'-*endo*, RNA-like sugar pucker) and stabilizing the A-form of RNA duplex, have been used to avoid off-target effects enhancing the specific recognition by the RISC. The development of unlocked nucleic acids (UNA) [18,19], threoninol nucleic acids (aTNA) [20,21], and serinol nucleic acids (SNA) [22,23] (Figure 1) has demonstrated that also more flexible acyclic derivatives can increase stability towards nucleases and, in addition, the introduction of these modifications at certain positions of siRNA molecule may improve some pivotal biological properties such as the potency [18,24].

**Figure 2:**
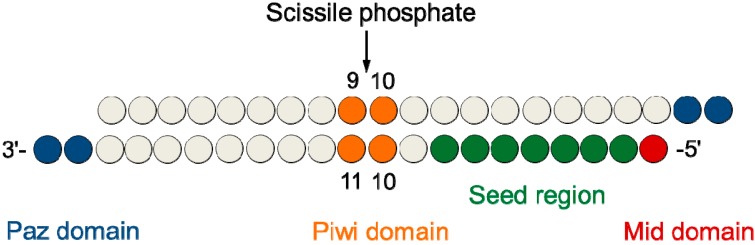
Schematic representation of a 21-mer siRNA duplex. The upper strand is the sense (**SS**), the lower strand is the antisense (**AS**) that guides the cleavage of the cognate mRNA. Only one of the two strands is retained into the Ago2 protein (the antisense), the sense strand is cut and degraded.

Likewise, thanks to chemical modifications, the RISC functionality and the role of the Ago2 domains (the core protein of the RISC), have been successfully unveiled [9,25,26]. Such findings allowed the designing of tailored siRNA molecules, avoiding some pivotal off-target effects like non-specific silencing seed-mediated, misloading of the sense strand, induction of interferon response, and saturation of the RNAi machinery. Specifically, it has been described that (i) the 5'-end phosphorylation of the antisense strand (Figure 2) is important for the recognition of the MID domain of the Ago2 protein and for the proper functionality of the siRNA molecule [27]; (ii) the stretch 2–8 of the antisense strand, called seed region, influences the miRNA-like silencing and that its extensive modification should lead to more specific silence [28]; (iii) the 3'-end overhang of the antisense strand is recognized by the PAZ domain and the presence of bulky modifications on positions 20 and 21 could affect the potency [29]; and (iv) the presence of modifications close to the cleavage site, especially at positions 10 and 11 of the antisense strand, may induce a strong impact in siRNA-mediated silencing [30,31,32].

Of special concern about RNA interference is the reduction of the off-target effects [33]. One important source of these off-target effects comes from the wrong selection of the antisense strand by RISC. The selection of the strand, that will guide the silence, depends on the relative thermodynamic stabilities of the two ends of the siRNA duplex. The strand with the less stable 5'-end is preferentially incorporated into the RISC [34,35]. For this reason, it has been described that the seed-region (positons 2–8 of the antisense strand) should be A-T rich, as the weaker base-pairing stability increases the probability of being selected by the RISC to form the silencing complex [36,37]. Here, as an extension of previous work done by our group [20], we evaluated the effect of acyclic L-threoninol nucleic acid (aTNA) at certain positions of the siRNAs. Specifically, we have concentrated our interest at positions 10 and 11 of the antisense strand and at position 2 of the sense strand. We studied the contribution of aTNA in slicing ability of the RISC and the possible correlation between the thermal stability of mismatches on the siRNA potency and the ON/OFF-target activity. Furthermore, we aimed to develop modified siRNAs able to block the sense strand activity.

## 2. Results and Discussion

### 2.1. Design and Thermodynamic Properties of siRNA Carrying L-Threoninol Monomers

In order to evaluate the effect of the introduction of L-threoninol modification on duplex stability, we compared the melting temperature of siRNAs bearing either a natural uridine or L-threoninol-thymine (**T^L^**) (Table 1). We designed 21 nt in length siRNA strands, directed against the *Renilla Reniformis* luciferase gene [15], and the **T^L^** modification was introduced at the 10th or 11th position of the antisense strand, as they are the positions that direct the cleavage of the target mRNA by Ago2 protein. The **T^L^**-modified RNA strands were synthesized using the corresponding phosphoramidite monomer [20]. The complementary RNA sense strands carrying all four natural bases (A, C, G, U) in front of the **T^L^** modification (9th or 10th position of sense strand) were also prepared to appraise the contribution of mismatches to the duplex stability and to study the base discrimination properties of the **T^L^** modification near the cutting site of Ago2 protein.

**Figure 3:**
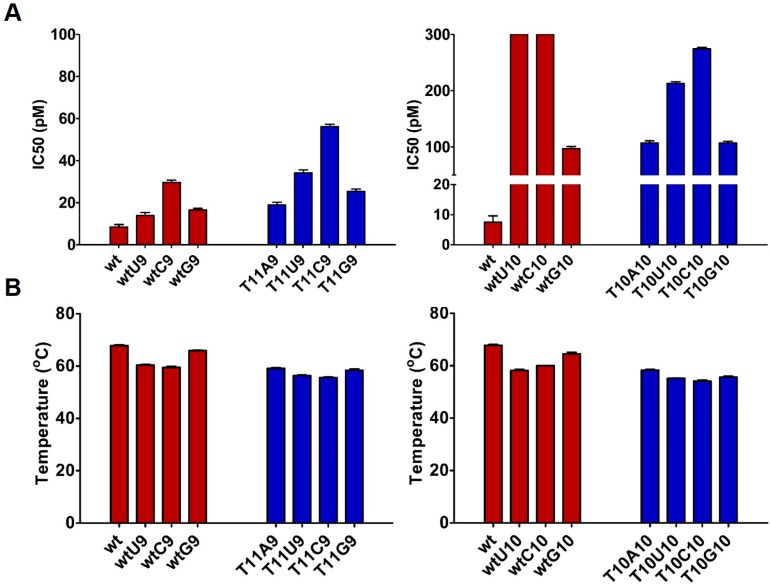
(**A**) IC_50_ assessments of siRNA molecules. To achieve the IC_50_ values, HeLa cells were co-transfected with psiCHECK2 (**AS**) reporter and decreasing amounts (1 nM, 0.3 nM, 60 pM, 16 pM, 8 pM and 2 pM) of siRNA molecules. Luminescence was evaluated 24 h after transfection. *n* = 3 ± SD; (**B**) Plot of siRNAs melting temperature used in luciferase study. *n* = 3 ± SD.

All the possible perfect matched and mismatched siRNA duplexes were annealed and thermal denaturation curves were recorded. Melting temperatures (T_m_) of siRNA duplexes are reported in Table 1. Results obtained from thermal denaturation experiments give us precious information about the hybridization properties of the L-threoninol-thymine modification. The introduction of L-threoninol-thymine modification in the middle of RNA duplexes impacted heavily on the melting temperature. We observed that a single L-threoninol-thymine modification, placed either at positions 10 or 11, induced a decrease on the melting temperature of 8.7 and 9.5 °C with respect to a perfectly matched RNA duplex. Then, we evaluated the impact of all possible mismatches facing either natural uridine or the **T^L^** modification. Thermal denaturation experiments revealed that, in the case of mismatches facing a natural uridine, the reduction of melting temperature depends on the nature of the mismatch (Table 1 and Figure 3B). The pyrimidine:pyrimidine mismatches (U:U and U:C) severely affected the stability of the RNA duplex and the melting temperature decreased in the range of about 7–9 °C. Conversely, the pyrimidine:purine (U:G) mismatch (wobble base pair) [38], was characterized by a small variation of the melting temperature: 2.1 and 3.5 °C difference with respect to the natural U:A base pair.

Unlike the natural uridine, the melting temperature of RNA duplexes, containing mismatches facing **T^L^**, decreased homogenously (in the range of 3 °C), with respect to the perfect matches siRNA containing **T^L^** monomer. The formation of the base pair (**T^L^**:**G**) had a positive effect on the melting temperature, which was quite similar to the perfect matched RNA bearing **T^L^**. Notably, the **T^L^** modification suffers the effect of the presence of mismatches less. Even in the presence of hard mismatches such as pyrimidine:pyrimidine pairs, the decrease of the melting temperatures was not as drastic as noted in the case of a natural uridine.

The observed weaker T_m_ variation should probably proceed from a marked capacity of accommodation within the double-helix in presence of any mispair. The lack of sugar constraint makes the **T^L^** modification sufficiently flexible to adjust its conformation into the duplex and be unable to discriminate among different mismatches.

**Table 1:** Design and properties of siRNA targeting *Renilla* luciferase gene.

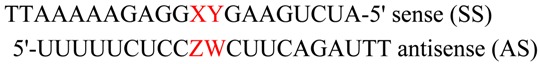

	Antisense ..ZW..	Sense ..XY..	IC_50_ (pM) ± SD	T_m_ (°C) ± SD	ΔT_m_ (wt)	ΔT_m_ (Parent)
wt	..UU..	..AA..	9.6 ± 0.5	67.8 ± 0.3	--	--
wtU9	..UU..	..AU..	15.4 ± 0.7	60.4 ± 0.2	7.6	7.6
wtC9	..UU..	..AC..	30.7 ± 0.6	59.5 ± 0.4	8.5	8.5
wtG9	..UU..	..AG..	15.8 ± 0.2	65.9 ± 0.2	2.1	2.1
wtU10	..UU..	..UA..	No active	58.2 ± 0.4	9.6	9.6
wtC10	..UU..	..CA..	No active	60.0 ± 0.1	7.8	7.8
wtG10	..UU..	..GA..	101 ± 0.7	64.5 ± 0.6	3.5	3.5
T10A10	..T^L^U..	..AA..	111 ± 0.8	58.3 ± 0.3	9.5	--
T11A9	..UT^L^..	..AA..	20.2 ± 0.6	59.1 ± 0.3	8.7	--
T10U10	..T^L^U..	..UA..	216 ± 0.6	55.2 ± 0.1	12.6	3.1
T10C10	..T^L^U..	..CA..	277 ± 0.9	54.1 ± 0.4	13.7	4.2
T10G10	..T^L^U..	..GA..	110 ± 0.9	55.7 ± 0.3	10.8	1.3
T11U9	..UT^L^..	..AU..	35.6 ± 0.4	56.4 ± 0.2	11.4	2.7
T11C9	..UT^L^..	..AC..	57.3 ± 0.5	55.6 ± 0.2	12.2	3.5
T11G9	..UT^L^..	..AG..	26.5 ± 0.8	58.4 ± 0.5	9.4	0.7

### 2.2. Impact of Mismatches and/or L-Threoninol Modifications on the Silencing Activity of siRNAs

In 2010 Maier and co-workers reported that the modulation of the thermal stability can enhance the potency of siRNA molecules [39]. The destabilization of the siRNA duplex could be achieved by the introduction of chemical modifications or on account of base pair mismatches [40,41]. The melting data stressed the destabilizing effects of the **T^L^** presence on the siRNA duplex (Table 1). Thus, by replacing the 10th and 11th positions of the antisense strand with **T^L^**, we want to investigate the impact of the modification on the cleavage site functionality and possible correlations between the duplex destabilization and the siRNA potency. The silencing activities (IC_50_) of the previously described siRNA duplexes are shown in Table 1. Luciferase experiments revealed that the modified siRNA at the 11th position (**T11A9**) discloses better activity (20.2 pM) than the one modified at the 10th position (**T10A10**, 111 pM), with respect to the activity of unmodified siRNA (**wt**, 9.6 pM). The comparison of IC_50_ activities defines more clearly the impact of the presence of **T^L^** on the silencing ability: only 2-fold change has been observed in the case of modification at the 11th position and around 10-fold change in presence of the 10th position modification (Table 1). These data allow us to conclude that the central positions of the antisense strand have a crucial impact on the silencing ability of siRNAs, likely due to alterations on the slicer activity of the Ago2 protein.

Next, we evaluated the silencing ability of different siRNAs carrying central mismatches at the 9th and 10th positions of the sense strand facing either natural uridine or **T^L^** residue. The introduction of mismatched base pair at central positions, locally destabilizing the RNA duplex, should entail better silence activities of the mismatched siRNAs by the rule “higher duplex destabilization leads to increase potency” [42].

Comparing the silence ability, among the **wtU9**, **wtC9**, and **wtG9** siRNAs carrying different mismatches at the 9th position of the sense strand and natural uridine on the antisense strand, we noted that the **wtU9** and **wtC9** siRNAs, although they own the lowest T_m_ (ΔT_m_ = 7.6 and 8.5 °C respectively), exhibited the same or worse silence activity with respect to the **wtG9** siRNA, characterized by the smallest change in T_m_ (ΔT_m_ = 2.1 °C). Thus, the correlation between thermal stability and siRNA potency is not so direct. In our hands, the siRNA duplexes with lower melting temperature exhibited worse silencing activities. The type of mismatch (U:U; U:C; U:G) influences the extent of the decrease. At the 9th position of the sense strand, the mismatch U:C (**wtC9**) affects more the siRNA activity respect to U:U (**wtU9**) and U:G (**wtG9**) mismatches. Indeed, the activity of **wtU9** (15.4 pM) and **wtG9** (15.8 pM) is almost preserved (**wt** = 9.6 pM), whereas the activity of **wtC9** (30.7 pM) revealed a larger reduction of potency (three-fold reduction). Considering the **T^L^**-modified siRNAs (**T11U9**, **T11C9**, and **T11G9**), although the potency is slightly affected with respect to natural mismatched siRNAs, the general silencing trend is maintained. The substitution **T^L^**:C impacted deeply on silencing, whereas the **T^L^**:U and **T^L^**:G mismatches disclosed better activity. Here too, the **T11G9** siRNA, characterized by the presence of the base pair **T^L^**:G, is associated with an activity (26.5 pM) quite similar to the parent **T11A9** (20.2 pM). Furthermore, depending on the position of the mismatches, the activity either decreased slightly (**wtU9**, **wtC9**, **wtG9**, **T11U9**, **T11C9**, and **T11G9**) or is strongly diminished (**wtC10**, **wtU10**, **wtG10**, **T10U10**, **T10C10**, and **T10G10**) with respect to the activity of the perfect match siRNA (**wt**). In the case of **wtU9**, **wtC9**, and **wtG9** siRNAs, the silencing activity was reduced between two- and three-fold (15.4, 30.7, 15.8* versus* 9.6 pM), whereas **wtC10** and **wtU10** siRNAs were inactive and the **wtG10** siRNA was 10-fold less potent than the perfectly matched (**wt**). Paying attention to the **T^L^**-modified siRNAs at the 10th position, we noted that the silencing of mismatched siRNAs with pyrimidine:pyrimidine mismatches (**T10U10** and **T10C10**) is retained to some extent. Again looking to parent activity, the siRNA potency is retained in presence of the **T^L^**:G bulge (**T10G10**), in which, probably, the natural geometry of the RNA duplex is preserved. These observations are consistent with previous reports claiming the better tolerance of the RISC for wobble base pairing [43,44]. As previously described [45], the presence of central mismatches at positions 9 and 10 of the sense strand impairs gene silencing (Figure 3A). The integrity of the A-form of the RNA double helix, especially in the proximity of the cleavage site, is a pivotal feature for the proper functionality of the RISC [46]. Thus, more than the melting temperature and so the destabilization of the duplex, the siRNA potency should be linked to the local distortion around the cleavage site. The correlation between duplex integrity and siRNA activity is adequately supported by the data arisen from luciferase experiments. Irrespective of position, siRNAs carrying the U:G wobble base pair, closely resembling a Watson-Crick base pair, disclosed the better activity among all mismatched siRNAs (Figure 3A). Moreover, Patel and co-workers [47] found that contiguous base pairing, along the segment starting from the positions 1–10 of the antisense strand, is necessary for the proper activity of RNase H-mediated cleavage of the Ago2 protein. For this reason, the alteration of base pairing due to the presence of mismatches at the 10th position of the sense strand impacts more heavily on the silencing, especially compared to siRNAs bearing a mismatch at the 9th position. Opposing views regarding the silencing mechanism of central mismatched dsRNA claimed that central bulges near the cleavage site can promote or hinder a slicer-dependent unwinding [48,49]. The presence of L-threoninol modification at position 10 of the antisense strand could make cleavable the sense strand, boosting a faster slicing-dependent unwinding, or affecting the local thermodynamic base pair stability, which would promote a slicer-independent unwinding. However, considering the melting temperature of **T10U10** and **T10C10** siRNAs, the lowest among all the synthetized siRNAs (55.2 and 54.1 °C, respectively) (Table 1), we thought that a bypass mechanism such as a slicer-independent unwinding should be at the basis of the slight retrieval activity.

### 2.3. Central Mismatched siRNA: Trying to Bias the Silence

**Figure 4:**
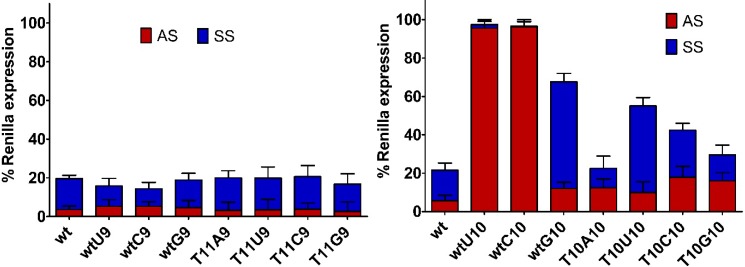
Silencing activities of antisense strand (**AS**) and sense strand (**SS**) of mismatched siRNAs at position 9 (left panel) and position 10 (right panel) bearing either natural uridine (**wt**) or L-threoninol-thymine (**T^L^**). To assess the on-target and off-target effects, 1 nM of the indicated siRNAs were co-transfected with psiCHECK2 reporters (**AS** or **SS**, **SSU9**, **SSC9**, **SSG9**, **SSU10**, **SSC10** and **SSG10**, respectively) in HeLa cells. Mock transfection was set as 100%. *n* = 3 ± SD.

In 2009, a report from Okamura* et al.* [50] stressed the importance of miRNAs duplex pairing status at position 9 and 10 during the strand selection of *Drosophila* Ago2 protein. Presence of mismatches at these positions drives a predominant selection for one strand of the miRNA molecule. More recent study [51] also confirmed that central mismatch at the 10th position of the antisense strand can bias the strand selection by the human Ago2 protein. Even if the strand selection by the Ago2 protein was not determined in this study, we wondered whether internal duplex structural features might affect the ON-/OFF-target activity, contributing to the design of functionally asymmetric siRNAs [52,53]. The individual activity of the two strands of a siRNA molecule can be adequately evaluated using a psiCHECK2 reporter system. Thus, eight different perfect sites for antisense strand (**AS**) and sense strand (**SS**; **SSU9**; **SSC9**; **SSG9**; **SSU10**; **SSC10**; **SSG10**) of the siRNA duplex (5' > 3') were inserted at the 3' untranslated region (3'-UTR) of the *Renilla* luciferase (Rluc) gene. As previously, central mismatches were produced substituting the A with U, C and G on positions 9 and 10 of the sense strand (facing the 11 and 10 positions of the antisense strand, respectively). The silence activity of the sense and antisense strand of these two sets of siRNAs were separately evaluated (Figure 4). Mismatched siRNAs at position 9 of the sense strand (**wtU9**, **wtC9**, **wtG9**, **T11U9**, **T11G9**. and **T11C9**) exhibited no substantial difference between the ON-/OFF-target silencing and were completely comparable to the perfectly matched siRNA (**wt**) (Figure 4). At first glance, the group of mismatched siRNAs at position 10 was globally characterized by reduced ON-activity (Table 1). Remarkably, the **wtU10** and **wtC10** siRNAs were not active, whereas the similar siRNA carrying **T^L^** modification (**T10U10** and **T10C10**), kept some degree of silencing (Figure 4 and Table 1). Unfortunately, the absence of activity of **wtU10** and **wtC10** siRNAs precluded further analysis on their ability of discrimination between ON-/OFF-target silencing. Unlike the mismatched siRNAs at the 9th position, L-threoninol modified siRNAs carrying mismatches at position 10 disclosed some asymmetry between the ON-/OFF-target silencing, in fact, siRNAs **T10U10**, **T10C10**, and **T10G10** seem to follow a pattern dependent on mismatch type. In detail, the presence of hard mismatches, such as pyrimidine:pyrimidine pairings (**T10U10** and **T10C10**) correspond to a certain bias towards the ON-target silencing. The **T10G10** siRNA, characterized by mild pyrimidine:purine mismatch (wobble base pair), disclosed the worst relationship between ON-/OFF-target silencing. Of note, siRNAs with mild pyrimidine:purine mismatch at position 10 (**wtG10** and **T10G10**), even maintaining the same ON-target potency (101 pM and 110 pM, respectively) revealed different OFF-target silencing. The presence of the L-threoninol unit instead of a natural uridine confers worse OFF-target silencing. In conclusion, not only the RNA helix geometry but also the mismatch position seems to be decisive for the degree of the ON-/OFF-target silencing.

### 2.4. Central L-Threoninol Modified siRNAs act through an Ago2-Mediated Mechanism

**Figure 5:**
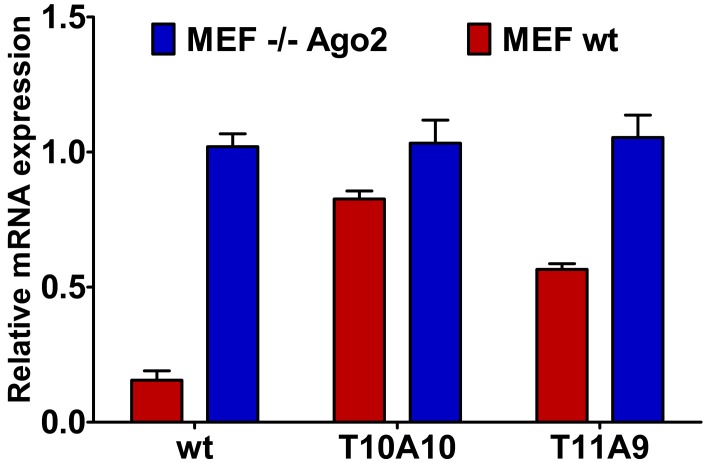
*Renilla* mRNA reduction in MEF^wt^ and MEF^Ago2−/−^ cells. 1 nM of unmodified (**wt**) and modified (**T10A10** and **T11A9**) siRNAs were co-transfected with psiCHECK2 (**AS**) reporter. After 24 h, cells were harvested for RNA extraction and qRT-PCR measurement. *n* = 2 ± SD.

In order to demonstrate that the silencing of central L-threoninol modified siRNAs (**T10A10** and **T11A9**) depends on the action of the slicer protein Ago2, we performed some RNAi experiments involving MEF^wt^ and MEF^Ago2−/−^ cells (Figure 5). In MEF^Ago2−/−^ cells, no *Renilla* mRNA decrease neither with unmodified (**wt**) nor with modified (**T10A10** and **T11A9**) siRNAs was observed. Conversely, in MEF^wt^ cells, both modified (**T10A10** and **T11A9**) siRNAs disclosed only moderate reduction of *Renilla* mRNA with respect to the **wt** siRNA. The presence of L-threoninol-thymine hindered the proper action of the Ago2 protein. Here too, the 10th position of the antisense strand proved to be more sensitive to modification than the 11th position. Noteworthy, the activities of modified (**T10A10** and **T11A9**) siRNAs reflected the IC_50_ values assessed by the luciferase assay (Table 1).

### 2.5. Single-Stranded siRNAs Experiments

**Figure 6:**
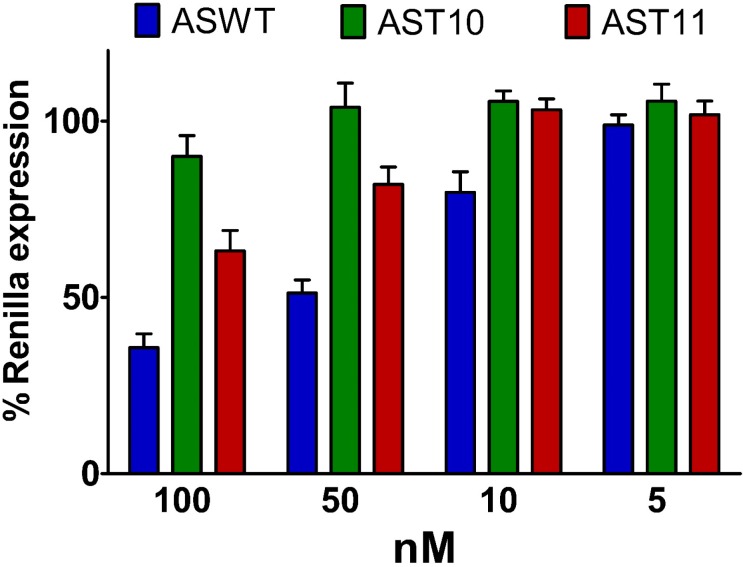
Plot of antisense ss-siRNAs activities of natural (**ASWT**) and **T^L^**-modified ss-siRNAs (**AST10** and **AST11**). Variable amounts of siRNAs (100 nM, 50 nM, 10 nM and 5 nM) were co-transfected with psiCHECK2 (**AS**) reporter in HeLa cells. 24 h post-transfection the luminescence were measured. *n* = 3 ± SD.

Finally, in order to assess the unambiguous contribution of the **T^L^** modification to the silence activity, some antisense single-stranded siRNA (ss-siRNA) assays were performed [20]. As depicted in Figure 6, the presence of the **T^L^** modification hindered the silencing, especially if compared to the unmodified ss-siRNA. Of note, the ss-siRNA modified with **T^L^** at the 10th position (**AST10**), even at the greatest dose transfected (100 nM), showed a pronounced reduction on activity with respect to both **T^L^**-modified at the 11th position (**AST11**) and the unmodified one (**ASWT**). The **AST11** ss-siRNA retained to a certain extent the silencing ability, although it is 4-fold less potent than the **ASWT**. Probably, the alteration of the catalytic site, given by the presence of the **T^L^** modification, should be the cause of the reduced or abolished silencing activity. Thus, the presence of the **T^L^** modification could alter the optimal geometry of the active site necessary to the mRNA cleavage. Taking together the data that emerged from MEF^Ago2−/−^ and ss-siRNA assays, we gather that this phenomenon is probably due to less efficient cleavage of the target mRNA. Besides conformational alteration at the cleavage site, variation in hybridization properties of the ss-siRNA with the cognate mRNA may also have an influence on activity.

### 2.6. Silencing Asymmetry and “Strand-Blocking” Effect of **T^L^** Modification

**Figure 7:**
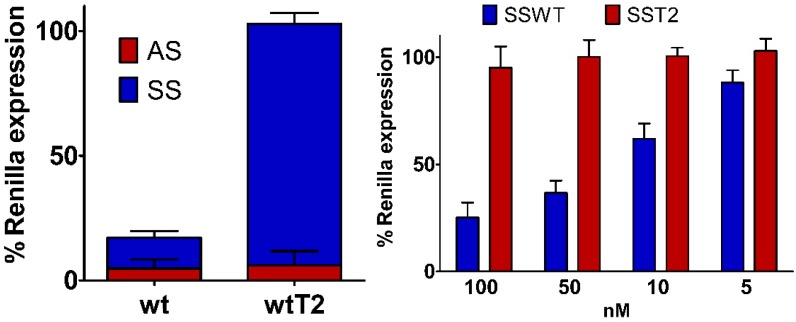
(left panel) ON-/OFF-target silencing of L-threoninol modified siRNA. Unmodified (**wt**) and modified at position 2 of the sense strand (**wtT2**) siRNAs, at final concentration of 1 nM, were co-transfected with psiCHECK2 sensors (**AS** and **SS**) in HeLa cells. Mock transfection was set as 100%. *n* = 3 ± SD. (right panel) Silencing activities of unmodified (**SSWT**) and position 2 **T^L^**-modified (**SST2**) sense ss-siRNA in HeLa cells. Luminescence were determined 24 h after the co-trasfection of decreasing concentrations of ss-siRNAs (100 nM, 50 nM, 30 nM and 5 nM) with psiCHECK2 (**SS**) reporter. Mock trasfection was set as 100%. *n* = 3 ± SD.

Results achieved from mismatched siRNA experiments suggested us that, in contrast to other reports [42], the mismatches introduction at central positions of siRNA molecules did not imply satisfactory bias between the ON-/OFF-target silencing (Figure 4). So, central structural parameters are pivotal for the silencing effectiveness but not for widening the silencing asymmetry. In order to estimate the rules governing silencing asymmetry of siRNA molecules, we thought to investigate the gene-silencing effects of the introduction of some flexible unit at the 5'-end of the sense strand as the L-threoninol modification. Thus, we inserted a single L-threoninol unit at position 2 (from the 5'-end) of the sense strand of a siRNA molecule (**wtT2**) (Table 2). The **wtT2** siRNA revealed a potency (13.4 pM) resembling that observed with the **wt** siRNA (9.6 pM), and the melting temperature subtly changed (ΔT_m_ = 1.1 °C) compared to unmodified siRNA (**wt**) (Table 2). The presence of only one L-threoninol unit near the 5'-end of the sense strand completely shifted the balance towards the ON-target activity (Figure 7). Furthermore, important parameters such as the potency and the duplex stability were preserved. Moreover, to examine the mechanisms underlying the silencing asymmetry obtained by the **wtT2** siRNA, we compared the activities of unmodified (**SSWT**) and position 2 L-threoninol modified (**SST2**) sense ss-siRNAs (Figure 7). The **SST2** ss-siRNA showed no notable silencing ability, whereas the **SSWT** ss-siRNA displayed a dose-dependent inhibition. Taking together the outcomes evinced from double-stranded and single-stranded siRNA experiments (Figure 7), we can affirm that the L-threoninol unit, impairing the gene-silencing ability of the modified sense strand, acted as “strand-blocking” modification. Probably, structural perturbations occurring between the sense seed region and the target mRNA totally blocked the sense strand activity [36]. The “strand-blocking” effect is even stronger in comparison with the impairment obtained by UNA modification [24]. Additionally, compared to other reports that achieved the same “strand-blocking” effect introducing various modifications on both siRNA strands [22], we completely abolished the sense strand-mediated gene-silencing activity with the introduction of a single L-threoninol modification.

**Table 2:** Sequence, potency and duplex stability properties comparison between unmodified and L-threoninol terminal modified siRNA.

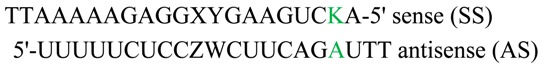

	Antisense ..A..	Sense ..K..	IC_50_ (pM) ± SD	T_m_ (°C) ± SD	Δ T_m_ (wt)
wt	..A..	..U..	9.6 ± 0.5	67.8 ± 0.3	--
wtT2	..A..	..T^L^..	13.4 ± 0.3	66.7 ± 0.3	1.1

## 3. Experimental Section

### 3.1. RNA Synthesis

RNA strands containing no modifications and a **T**^L^ unit at positions 10 and 11 were synthesized on the 0.2-μmol scale using LV200 polystyrene supports. All oligonucleotides were synthesized on an Applied Biosystems 394 synthesizer (Foster City, CA, USA) using commercially available reagents (Fluka and Sigma-Aldrich, Quimica S.A., Tres cantos, Madrid, Spain) and 2'-O-TBDMS-5'-O-DMT-protected phosphoramidites (A^Bz^, G^dmf^, C^Ac^ , and U) (Link Technologies, Glasgow, Scotland, UK). The synthesis procedure of the L-threoninol-thymine phosphoramidite was already described [20]. The coupling time was 15 min and the coupling yields of natural and modified phosphoramidites were >97% in DMT-ON mode. SiRNAs previously described by Terrazas* et al.* [15] were used to design siRNA duplexes against the *Renilla* gene.

### 3.2. Deprotection and Purification of Unmodified and Modified RNA Oligonucleotide

Every solid support was treated at 55 °C for 1 h with 1.5 mL of NH_3_ solution (33%) and 0.5 mL of ethanol. Then, the suspension was cooled to room temperature; the supernatant was transferred into a clean tube and subsequently evaporated to dryness using a Speedvac concentrator. The obtained residue was dissolved in 1 M TBAF in THF (85 μL per 0.2 μmol resin) and incubated for 15 h at room temperature. Finally, 1 M triethylammonium acetate (TEEA) and water were added to the solution (0.2 μmol synthesis: 85 μL of 1 M triethylammonium acetate (TEEA) and 330 μL water). Oligonucleotide desalting procedure was conducted on NAP-5 columns using water as eluent and evaporated to dryness. The purification of oligonucleotides was carried out by HPLC (DMT-ON). Column: Nucleosil 120-10 C18 column (250 × 4 mm). Solvent A: 5% ACN in 0.1 M aqueous TEAAc (pH = 7) and solvent B: 70% ACN in 0.1 M aqueous TEAA (pH = 7). Flow rate: 3 mL/min. Conditions: 20 min linear gradient from 15% to 80% B and 5 min 80% B. The collected pure fractions were evaporated to dryness and then treated with 1 mL of 80% AcOH solution and incubated at room temperature for 30 min. The deprotected oligonucleotides were desalted on NAP-10 column using water as eluent.

### 3.3. SiRNA Preparation

SiRNA duplexes were annealed with equimolar ratios of the sense and the antisense strands in siRNA suspension solution (100 mM KOAc, 30 mM HEPES-KOH and 2 mM MgCl_2_, pH 7.4) at final concentration of 20 μM. Duplexes were heated at 95 °C for 5 min and slowly cooled to 4 °C.

### 3.4. Thermal Denaturation Studies

Melting curves of duplex RNAs were performed following change of absorbance at 260 nm* versus* temperature. Samples were heated from 20 °C to 80 °C, with a linear temperature ramp of 0.5 °C/min in a JASCO V-650 spectrophotometer (JASCO, Easton, MD, USA) equipped with a Peltier temperature control. All the measurements were repeated in triplicate, both the heating and cooling curves were measured. Buffer condition: 100 mM KOAc, 30 mM HEPES-KOH, 2 mM MgCl_2_, pH 7.4, [oligonucleotide] = 1 M.

### 3.5. Cells

HeLa cells (ATCC), MEF^wt^ cells (ATCC) and MEF^Ago2−/−^ cell lines (a kind gift of Dr. O’Carroll [54]) were maintained in monolayer culture at exponential growth in high-glucose Dulbecco modified Eagle medium (DMEM) (Gibco, Life Technologies, Carlsbad, CA, USA) supplemented with 10% heat inactivated fetal bovine serum (Gibco, Life Technologies, Carlsbad, CA, USA) and 1× penicillin/streptomycin solution (Gibco, Life Technologies, Carlsbad, CA, USA). All cell lines were incubated at 37 °C in a humidified environment with 5% CO_2_ and periodically checked for the presence of mycoplasma contamination. Cell viability was monitored by Trypan Blue exclusion assay and was higher than 95% in all experiments.

### 3.6. PsiCHECK2 on-/off-Target Reporters

To construct the on-target (**AS**) and off-target (**SS**; **SSU9**; **SSC9**; **SSG9**; **SSU10**; **SSC10**; **SSG10**) reporters, 5' phosphorylated DNA sequences (Sigma-Aldrich, Quimica S.A., Tres cantos, Madrid, Spain) corresponding to the antisense strand target (5'-TCGAGTCAAATCTGAAGAAGGAGAAAAAGC and 5'-GGCCGCTTTTTCTCCTTCTTCAGATTTGAC) and sense-strand target (**SS**: 5'-TCGAGATTTTTCTCCTTCTTCAGATCGTGGC and 5'-GGCCGCCACGATCTGAAGAAGGAGAAAAATC; **SSU9**: 5'-GGCCGCCACGATCTGAAGTAGGAGAAAAATC and 5'-TCGAGATTTTTCTCCTACTTCAGATCGTGGC; **SSC9**: 5'-GGCCGCCACGATCTGAAGCAGGAGAAAAATC and 5'-TCGAGATTTTTCTCCTGCTTCAGATCGTGGC; **SSG9**: 5'-GGCCGCCACGATCTGAAGGAGGAGAAAAATC and 5'-TCGAGATTTTTCTCCTCCTTCAGATCGTGGC; **SSU10**: 5'-GGCCGCCACGATCTGAAATTGGAGAAAAATC and 5'-TCGAGATTTTTCTCCATCTTCAGATCGTGGC; **SSC10**: 5'-GGCCGCCACGATCTGAAATCGGAGAAAAATC and 5'-TCGAGATTTTTCTCCGTCTTCAGATCGTGGC; **SSG10**: 5'-GGCCGCCACGATCTGAAATGGGAGAAAAATC and 5'-TCGAGATTTTTCTCCCTCTTCAGATCGTGGC) of the synthesized siRNAs were annealed and inserted into the *XhoI* and *NotI* sites of the psiCHECK2 plasmid (Promega, Madrid, Spain). The correct insertion of the sequences was confirmed by sequencing.

### 3.7. Transfection and Luciferase Assay

For double-stranded and single-stranded siRNA luciferase assay, HeLa cells were plated in 24-well tissue culture plates at density of 1 × 10^5^ cells per well 24 h before transfection. In dose response, ON-/OFF-target assessment and single-stranded siRNAs experiments, 1μg of psiCHECK2 (**AS**) or psiCHECK2 (**SS**; **SSU9**; **SSC9**; **SSG9**; **SSU10**; **SSC10**; **SSG10**) and siRNAs at different concentrations were co-transfected using Lipofectamine 2000 (Life Technologies, Carlsbad, CA, USA) in accordance with the manufacturer’s instructions. The inhibitory effect of siRNAs on *Renilla* protein expression was measured on lysates collected 24 h after transfection using the Dual-Luciferase Reporter Assay System (Promega, Madrid, Spain) and a SpectraMax M5 luminometer (Molecular Devices, Sunnyvale, CA, USA). The ratios of *Renilla* luciferase (hRluc) to *Photinus* luciferase (hluc+) protein activities were normalized to mock transfection and the mock activity was set as 100%.

### 3.8. Ago2-Mediated Silencing Assay

MEF^wt^ and MEF^Ago2−/−^ cells were plated in 24-well tissue culture plates at a density of 0.8 × 10^5^ cells per well 24 h before transfection. Co-transfection of AS reported plasmid and different siRNAs molecules (**wt**, **T10A10** and **T11A9**) at concentration of 1 nM was performed with lipofectamine LTX (Life Technologies, Carlsbad, CA, USA) in accordance with the manufacturer’s protocol for MEF. After 24 h, the samples were harvested for RNA extraction.

### 3.9. Single-Stranded siRNA 5'-End Phosphorylation

Before transfection, 300 pmol of single-stranded antisense (**ASWT**; **AST10**; **AST11**) and sense (**SSWT**; **SST2**) siRNAs (ss-siRNAs) were incubated for 90 min at 37 °C with 100 mM of ATP and T4 Polynucleotide kinase (3'phosphatase minus) (New England Biolabs, Ipswich, MA, USA), then for 30 min at 65 °C to inactivate the enzyme, following the manufacturer’s instructions.

### 3.10. Isolation of RNA and RT-qPCR

Total RNA was isolated from MEF^wt^ and MEF^Ago2−/−^ with TRIzol reagent (Invitrogen, Carlsbad, CA, USA). Then, extracted RNA was quantified by NanoDrop (Thermo Scientific, Waltham, MA USA). Of each RNA sample, 2.5 µg was treated with DNase I [DNase I (RNase free) New England Biolabs] following the manufacturer’s instructions. Then, the reverse transcription reaction, 0.5 µg of total RNA, was carried out with random hexamer primers and Revertaid H minus RT enzyme (Thermo Scientific) according to the manufacturer’s instructions. First, strand cDNA was subsequently diluted 4 times in nuclease-free water before the addition of 1 µL of resulting cDNA to the real-time mixture. Real-time PCR was accomplished in a total volume of 20 µL, using Maxima SYBR Green protocol (Thermo Scientific) following the manufacturer’s instructions. The reference gene GADPH was used as the internal control. *Renilla* silencing was calculated and represented as 2^−∆∆Ct^ method. All primer pairs were purchased from Sigma-Aldrich and Primer-Blast was used as the primer designing tool [55]. GAPDH Fwd: 5'-TGCACCACCAACTGCTTAG; GAPDH Rev: 5'-GATGCAGGGATGATGTTC; hRluc Fwd: 5'-GGGCGAGAAAATGGTGCTTG; hRluc Rev: 5'-GCCCTTCTCCTTGAATGGCT.

### 3.11. Statistical Analysis

Statistical analysis was performed using GraphPad Prism software (GraphPad, San Diego, CA, USA). IC_50_ determination was performed using non-linear regression analysis (log [inhibitor]* vs.* normalized response).

## 4. Conclusions

In conclusion, the L-threoninol-thymine modification, thanks to the lack of the sugar constraint, allows us to pay specific attention to the nucleobase contribution and the influence of the ribose ring on the active site of RISC. Also, it proved to be an extremely useful tool, not only for the understanding of its base pairing and ON-/OFF-target silencing properties, but also for dissecting some important issues regarding the sense strand activity. Furthermore, the “strand-blocking” effect achieved by the introduction of a single L-threoninol unit might be exploited for tailored design of functionally asymmetric siRNA molecules and it also renders its siRNA synthesis time and its cost effectiveness.
